# The Potential of Self-Management mHealth for Pediatric Cystic Fibrosis: Mixed-Methods Study for Health Care and App Assessment

**DOI:** 10.2196/13362

**Published:** 2019-04-18

**Authors:** Antonio Martinez-Millana, Annabel Zettl, Jacqueline Floch, Joaquim Calvo-Lerma, Jose Luis Sevillano, Carmen Ribes-Koninckx, Vicente Traver

**Affiliations:** 1 Instituto ITACA Universitat Politècnica de València Valencia Spain; 2 Unidad Mixta de Reingeniería de Procesos Sociosanitarios Instituto de Investigación Sanitaria del Hospital Universitario y Politecnico La Fe Valencia Spain; 3 Youse GmbH München Germany; 4 SINTEF Trondheim Norway; 5 Instituto de Investigación Sanitaria La Fe Valencia Spain; 6 Department of Architecture and Technology of Computers University of Seville Seville Spain

**Keywords:** mHealth, cystic fibrosis, health care systems, user requirements, apps, market analysis

## Abstract

**Background:**

Remote care services and patient empowerment have boosted mobile health (mHealth). A study of user needs related to mHealth for pediatric cystic fibrosis (PCF) identified the set of preferred features mobile apps should support; however, the potential use of PCF apps and their suitability to fit into PCF clinical management remains unexplored.

**Objective:**

We examine whether PCF holds potential for the implementation of mHealth care.

**Methods:**

The study is based on a literature review and qualitative analysis of content and was conducted in two parts: (1) we reviewed scientific and gray literature to explore how European countries manage PCF and conducted a qualitative study of 6 PCF units and (2) we performed a systematic review of apps available in the myhealthapps.net repository searching for cystic fibrosis (CF) management and nutrition apps, which we analyzed for characteristics, business models, number of downloads, and usability.

**Results:**

European CF routine care guidelines are acknowledged in most European countries, and treatments are fully covered in almost all countries. The majority of teams in CF units are interdisciplinary. With respect to the systematic review of apps, we reviewed 12 apps for CF management and 9 for general nutrition management in the myhealthapps.net directory. All analyzed apps provided functionalities for recording aspects related to the disease and nutrition such as medication, meals, measurements, reminders, and educational material. None of the apps reviewed in this study supported pancreatic enzyme replacement therapy. CF apps proved to be less appealing and usable than nutrition apps (2.66 [SD 1.15] vs 4.01 [SD 0.90]; *P*<.001, *z*-value: –2.6). User needs detected in previous research are partially matched by current apps for CF management.

**Conclusions:**

The health care context for PCF is a unique opportunity for the adoption of mHealth. Well-established clinical guidelines, heterogeneous clinical teams, and coverage by national health care systems provide a suitable scenario for the use of mHealth solutions. However, available apps for CF self-management do not cover essential aspects such as nutrition and education. To increase the adoption of mHealth for CF self-management, new apps should include these features.

**International Registered Report Identifier (IRRID):**

RR2-10.1136/bmjopen-2016-014931

## Introduction

Pediatric cystic fibrosis (PCF) is a childhood-onset inherited disease characterized by progressive lung and pancreas deterioration, leading to premature death. Estimations in the European Union are about 30,000 cystic fibrosis (CF) patients, with one new case per 1800 to 25,000 newborns depending on the region [1]. CF inception is based in the mutation of a gene encoding the CF transmembrane conductance regulator. Despite impressive advances relating the molecular basis of CF to organ-level disease, life expectancy and quality of life are still limited in CF [2]. The combination of early diagnosis and early intervention promises to slow down the progression and improve the clinical course of the disease [3].

The primary pillars of proper CF management include a continuum of care and treatment in which the following goals are highlighted: (1) maintaining lung functions as near to normal as possible by controlling respiratory infection; (2) providing nutritional support (ie, pancreatic enzyme supplements and an adapted and balanced diet) to achieve adequate nutritional status and growth [4]; and (3) complication prevention, detection, and management [5]. To achieve these goals, a multidisciplinary team of specialized and expert health professionals is required [6,7].

National public health systems and health insurance systems are in charge of supporting and covering PCF patient treatments. While the ways countries approach these costs differ significantly [8], care services provided are similar in many high-income countries—mainly through specialized units in university hospitals and specialized centers in which follow-up outpatient visits are scheduled at 2- to 3-month intervals for physical examination and specific laboratory tests [5,8].

CF management is time-consuming and requires a multidisciplinary approach [4]. The number and variety of treatments patients are prescribed complicate both follow-up and adherence [9]. Mobile technologies (eg, mobile phones, wearable devices) provide highly scalable new approaches to disease management [10] and have shown evidence on benefits for health maintenance, disease management, and prevention of complications [11]. Mobile phone use has been rapidly increasing worldwide [12], and more than 50% of mobile phone owners use their devices to obtain health information [13].

Research in mobile health (mHealth) has demonstrated intrinsic motivations to facilitate mHealth adoption and perceived risks to inhibit it [14]. In 2013, Rai et al [15] presented a framework for evaluating drivers of mHealth implementation and found that users (patients and doctors) were more favorable to adopting mHealth as a complement to (not a substitute for) face-to-face visits. This framework has been assessed more recently [16-18], showing beneficial results when patients are supported from the very early stages after diagnosis and treatment initiation and when the technology achieves medical credibility. Nevertheless, authors highlight the need for qualitative research to assess institutional factors that would improve the acceptance of mHealth [16-18].

With regard to CF, Hilliard et al [19] asked 16 patients to report experiences using mobile apps and identify potential features to support CF self-management. Participants expressed excitement and interest in potential future mHealth apps designed to facilitate CF management, but they also raised concerns about usability, usefulness, and security. In our research within the MyCyFAPP project [20] and in the context of a user-centered design approach [21], we interviewed 74 people including persons with CF and caregivers and health professionals with CF care expertise. This study indicated the readiness for self-management in the CF community in countries that provide well-functioning health services for CF care and identified a large diversity of user requirements and a need for personalization. In addition, we derived a set of potentially useful features in an mHealth app for CF: educational tools, enzyme dosage calculation, nutritional management, treatment organization and follow-up, health diary, practical guidelines for treatment, communication with doctors, and communication with peers.

Several studies analyze the features of apps and their impact on the self-management of diseases (eg, diabetes [22], multiple sclerosis [23], cardiovascular diseases [24], and asthma [25]). These studies identify strengths and weaknesses of current apps and the requirements for improving the support of chronic diseases in the mHealth paradigm. Mobile apps have the potential to improve clinical outcomes and increase attendance and adherence rates [11].

Therefore, given that digital tools may be helpful in the self-management of CF [11] and considering the latest results on the types of features that would be particularly helpful for PCF mobile health management [21], we examined the way forward for the implementation of mHealth care by analyzing how European health care systems approach PCF management and identifying the characteristics of currently available apps for CF management. The rationale was to contribute to the adoption of mHealth in the management of PCF by the identification of strengths and opportunities. Our approach addressed the self-management of PCF using mobile technologies and targeted teenagers, young adults, and parents of young children.

The research presented in this paper was conducted within the context of the MyCyFAPP European Project (grant agreement number 643806) [20]. MyCyFAPP is developing a self-management tool for PCF patients that allows for personalized and accurate control and monitoring of the disease.

## Methods

### Design of Study

#### Health Care and App Assessment

The approach for analyzing the context of CF management consisted of two parts connected by the research objective. In part 1, analysis of CF care and health care systems was oriented to discover how different European Union countries provide overall care related to PCF. This analysis included health care and pharmacological support to patients and families through national health systems and health insurances. In addition, we analyzed the organization and coverage of health care systems in the specific domain of CF and in particular their ability to involve mobile apps in the process of managing the disease. The study did not focus on individual organizations but rather took a national perspective. In part 2, analysis of available apps in the specific context of CF in the official market stores was conducted to understand which types of functionalities are already provided and whether they are being used in a clinical context.

#### Part 1: National Health Care Cystic Fibrosis Coverage Analysis

The evaluation of health care services has been considered and used in the medical literature for improving the quality of care delivery and its efficiency [26,27]. Kerem et al [28] proposed a set of key performance indicators based on staff, health services, and facilities so CF centers could be compared on annual basis.

In the proposed country-by-country analysis of health care systems, we aimed to discover and compare how countries are organized to provide care and support to PCF patients based on a subset of the indicators proposed by Kerem et al [28]. We included medical aspects such as the follow-up protocol, newborn screening for CF, and composition of health care teams in CF units as well as economical aspects such as the coverage of medication costs and type of services provided to patients.

The main sources used for analysis were the European Cystic Fibrosis Society (ECFS) Patient Registry Annual Data Report from 2016 [29] and reports from national health care systems. The ECFS report contains demographic and clinical data of 44,719 consenting CF patients from 89 CF units and centers in 31 countries. Criteria for selecting countries were based on the number of registered patients and the geographical distribution and proportion of children (patients aged younger than 18 years) as collected in the ECFS 2016 report. This selection included Spain, Italy, Portugal, the Netherlands, Belgium, France, Norway, Germany, and the United Kingdom (Table 1). The health care organization and management of PCF in these countries were analyzed using official reports and literature. Data were extracted using a structured script: prevalence of PCF, protocols and guidelines for PCF management and control, insurance model, and availability of newborn screening programs.

Analysis of specific PCF units relied on semistructured interviews with professionals involved in the management of PCF who signed the informed consent from the study that was approved by the ethical committees of the participant hospitals. Beyond the context analysis, we aimed at discovering the composition of the clinical teams involved in the care and follow-up of PCF patients, the number and type of professional roles, and the size of the population covered by each unit. Interviews and one group discussion included pediatricians from 6 PCF units participating in the MyCyFAPP project who agreed to participate and gave consent to the analysis and publishing of their responses.

**Table 1:** Extraction of the estimated coverage, registered patients, and proportion of pediatric cystic fibrosis for the countries included in the study [29].

Country	Estimated coverage^a^, %	Registered patients, n	Proportion of pediatric cystic fibrosis, %
Belgium	>90	1282	39
France	90	6713	44
Germany	>80	5738	41
Italy	95	5384	41
The Netherlands	98	1449	40
Portugal	>95	339	56
Norway	72	230	34
Spain	70	1995	54
United Kingdom	99	10,465	43

^a^Percentage of the total estimated cystic fibrosis population covered by the study.

**Observed characteristics and descriptions of information collected for each app.**

- **App promoters:** entities that designed, developed, funded, and launched app (full name, website, reference contact information)
- **Promoter types:** hospitals, cystic fibrosis units, enterprises and the type (pharma, information and communications technology-based, etc), patient associations, charities, research projects, and other types of entities or consortia
- **Promoter goals:** short description of the main benefits for the user and intention of the promoting entities offering the app (eg, for dissemination purposes, to make a business out of the features offered, to help cystic fibrosis unit patients achieve self-management goals)
- **Geographical reach:** available languages and geographical scope in which the app would work as expected
- **Business models:** how promoters make the app sustainable in economic terms. This field indicated if there is any revenue model or payment for the use of the app or if the app was financed by the entity for promotion or as a kind of free outcome for the cystic fibrosis community. If possible, a description of the type of funding of the app was provided (eg, research funds, donations, private investment, public investment)
- **Usability score:** assessment of the user experience. Three human-computer interaction experts evaluated the usability of the apps. They used an ad hoc questionnaire composed of 5 questions focusing on design, interaction, attractiveness, structure, and understandability, each of them graded using a 5-point Likert scale in which “1” meant a low score and “5” meant a high score
- **Type of language/vocabulary:** identification of the context of use of each app (eg, if app was oriented to medical aspects and therefore written in a very technical language, if app was focused on providing guidelines and used basic or plain knowledge). Target user: adult, child, or both
- **Available platforms and facts:** marketplace availability (Google Play for Android and App Store for iOS), number of downloads per platform (as an estimation of use, although downloading does not represent use of the app), number of reviews (indicating the number of people who were interested enough to make their own assessment about app), publication date of the app on each platform and last review date (to establish app age and how often it is updated), app version currently available (to estimate evolution and releases)

### Part 2: Rapid Review of Cystic Fibrosis and Nutrition Apps

The analysis of available mobile apps had the objective of reviewing solutions in the field of CF oriented to pediatric patients, families, and health professionals in the management of CF or nutrition. The rapid review considered predefined specific criteria, and the search was done on myhealthapps.net (MHA), the directory of health apps supported and reviewed by patient associations and key collaborators [26]. MHA is an indexing and searching engine for apps endorsed by patients and consumer health forums that includes apps from trustworthy developers. The identification of apps was done using the search engine at the MHA portal using the following keywords: “cystic fibrosis” and “health diary” for CF apps and “nutrition” or “food” for nutrition apps.

The inclusion criteria for apps were defined as having a (1) title or description referring to CF or (2) description referring to nutrition management linked to chronic diseases. Exclusion criteria were defined as (1) no intended use for chronic condition management and (2) not available in official app stores or repositories. Collected information included the (1) app creator, (2) app purposes, (3) identified business models, and (4) other relevant information to be considered as a reference for app evolution, sustainability, and fit into a health care system. A description of the characteristics collected in the study can be found in Textbox 1.

### Statistical Analysis

Data extracted from the systematic review of apps were compared between CF and nutrition apps. The independence of categorical variables was assessed by means of chi-square tests (alpha=.05), and continuous variables were assessed using the Wilcoxon Test (alpha=.05). Statistical calculations were done using built-in functions in Matlab R2018a (The MathWorks, Inc).

## Results

### Part 1: National Health Care Cystic Fibrosis Coverage Analysis

#### General Findings

Routine care was homogeneous in the 9 countries included in this review. Regular follow-ups met international guidelines, scheduling a full assessment of PCF patients each 2 or 3 months. Newborn screening was implemented in 8 of the 9 studied countries. Treatments were fully covered in almost all countries. Only Belgium and the Netherlands only partially funded expenses related to treatment. Generally, the type of staff involved in CF units and reference centers for CF included pediatricians, dietitians, psychologists, and nurses, but some units also included social workers, physical rehabilitators, and pharmacists.

#### Belgium

The Belgian health care system is based on national and private health facilities funded by a combination of social security contributions and health insurance funds. The national health system reimburses a large portion of medical expenses. For CF, a full assessment every 2 to 3 months with multidisciplinary medical care and treatment will be reimbursed. The budget is managed by the national health system. For CF, some medications (eg, Creon and fat-soluble vitamins) are fully reimbursed and some medications (eg, antibiotics) are partially reimbursed. Newborn screening for CF has not yet been implemented in Belgium [30].

#### France

The French health care system is financed by the national health insurance and national budget. Fees and insurance levels are determined by several factors (income, consumed goods and services). CF patients are considered disabled and are eligible for 100% reimbursement from the national health insurance system. Also, parents of children with CF are granted social rights related to the long-term care and support of the disease in the children. Newborn screening of CF is established in France [31].

#### Germany

Germany has a universal health care system funded by a combination of statutory health insurance and private health insurance. The system is decentralized with private practice physicians providing ambulatory care and independent, mostly nonprofit hospitals providing the majority of inpatient care. Hospitals in Germany are reimbursed by health insurance companies according to diagnosis-related groups, a flat rate system that defines reimbursement rates depending on the diagnosis. Newborn screening is implemented countrywide. Follow-up of CF patients is scheduled every 3 months in outpatient clinics with a more in-depth checkup recommended annually [32].

#### Italy

The national health service in Italy is universal and free of charge. The system is decentralized and divided into 14 local health agencies (*Azienda Sanitaria Locale*). Basic medical care (eg, general medicine, pediatric care, hospital admissions) is fully covered by the national system but for other types of specialized services (eg, outpatient consultations, laboratory tests, prostheses) patients usually must pay a low-cost fee determined by their health care region, income level, and subscription to a private insurance.

Patients with CF are exempt from paying for follow-ups according to a specific law (law 548/93, GU 30/12/1993, n°305). According to this law, patients with CF must be followed up at specialized CF centers, which provide multidisciplinary care (including gastroenterology and pulmonology services) and drugs at no cost for patients. Follow-up is generally scheduled every 2 to 3 months, but the frequency of visits depends on patient health status and can range from daily to every 6 months [33]. A national newborn screening program is implemented; therefore, in the great majority of the patients, first referral to a CF center occurs very early in life, soon after diagnosis [33].

#### The Netherlands

The Netherlands has a dual-level health care system in which primary and curative care (eg, general medicine, hospital admissions) are financed by a private mandatory insurance. These companies are obliged to provide a package with a defined set of insured treatments. This insurance covers 41% of all health care expenses. Secondary care (eg, long-term care, end-of-life care) is covered by social insurance funded by a national budget. Follow-up of CF patients is done at least every 3 months by a pulmonologist and at least once a year by the pediatric gastroenterologist [34]. Treatments and medical services for CF are fully covered. CF is included in the national newborn screening program [35].

#### Norway

Health care in Norway is divided into 4 regional health authorities under the responsibility of the Ministry of Health and Care. Health care services are funded by a national budget and partially covered by fees. There is a unique competence center for CF—the Norwegian Resource Center for Cystic Fibrosis (NSCF)—located in Oslo. When CF is diagnosed and treatment initiated, all patients are treated by the NSCF. The parents get training about nutrition, physiotherapy, and other important topics relevant to the patient. Later on, patients are treated by local hospitals and local physiotherapists (NSCF if they live in the southeast region). NSCF provides training to the persons responsible for providing health services locally. CF patients are followed up regularly (every 1 to 3 months depending on health status) by doctors and nutritionists at a local hospital in the region where the patient lives. All patients are normally followed up at NSCF once a year. Shared cost is set for medical supervision and medication with some services being fully covered (eg, physiotherapy). All costs related to screening, confirmation of diagnosis, further treatment, and travel costs to NSCF are covered by the public health insurance. CF newborn screening is included in the national health care system [36].

#### Portugal

The health care system in Portugal consists of 3 different coexisting systems: national, professional, and private insurance. The national system provides universal coverage and is split into 5 regional administrations for which the national Ministry of Health defines health policies. General patients have to pay a fee for the medical consultation; however, CF patients are exempt from these fees. CF patients are scheduled every 1 to 2 months depending on patient health status (it could range from daily to every 6 months) in the CF unit, which includes gastroenterology and pulmonology services. Follow-up includes dietitians (with a nutritional evaluation) and nurses (for adherence to medical therapy) [37]. All the costs related to CF medications are supported by the national health system. Newborn screening is implemented countrywide and includes CF [38].

#### Spain

The national health system in Spain is universal and free of charge. The national Ministry of Health coordinates the 17 regional health systems, but the regional systems are autonomous and define their own health policies. Spain has 22 regional CF centers, which are mainly linked to university hospitals [39]. PCF patients are followed up every 2 to 3 months with complete assessments by pediatric pneumologists and gastroenterologists [39]. Additional and more frequent visits are scheduled depending on the patient’s clinical condition. An annual in-depth evaluation is also the rule in most CF units. In the case of PCF, the health care system covers all medical and pharmacy costs. Medications are delivered to the patients through the Pharmacy unit of the hospital so patients with CF do not have any associated medication costs. Additionally, oral nutritional supplements are provided for free at the hospitals. In 2012, a newborn screening program was implemented in almost all maternity wards in the country.

#### United Kingdom

The National Health System (NHS) in the United Kingdom is universal and free of charge. It consists of a decentralized system divided in 4 geopolitical regions. The NHS has established a national guideline for the diagnosis, treatment, and follow-up of CF [40] for health care professionals in primary care, health care professionals in secondary care, providers of CF services, and practitioners in CF. CF prescription charges have been abolished entirely for people in Scotland, Northern Ireland, and Wales, but in England many people with CF still have to pay for their prescriptions. The Cystic Fibrosis Trust offers financial support for emergency care. Finally, the United Kingdom has deployed newborn screening countrywide [41].

#### Center-Level Analysis

In Multimedia Appendix 1, we show a comparison of the specific managerial and clinical organization of 6 European CF units that are participants in the MyCyFAPP project [20] and gave us access to information about their institutions. Among these centers, 4 of the 6 have a separate unit for managing PCF patients. The median number of pediatric patients (patients aged younger than 18 years) in CF units is 127. The center with the highest number of children is the University Hospital of Milan (399); the lowest is the *Associação Para Investigação, Desenvolvimento Da Faculdade De Medicina* of Lisbon (60).

The main difference among centers is the composition of clinical teams involved in PCF care and follow-up. All the interviewed units have roles for covering gastrointestinal, pulmonary, and nutritional aspects of the disease (adhering to current guidelines [4]), but we observe that one (*Ramon y Cajal Hospital* in Madrid) includes two endocrinologists. Two other differences are the inclusion of social workers and psychologists at the University Hospital of Milan, Erasmus University Medical Center, and KU Leuven Hospital and the inclusion of a pharmacologist at the KU Leuven Hospital.

Involved clinical staff is heterogeneous among and within countries; differences are not only country-specific but depend on specific constraints at a hospital/unit level, as we observe by comparing the two units in Spain (*La Fe* vs *Ramon y Cajal Hospital*).

### Part 2: Systematic Review of Cystic Fibrosis and Nutrition Apps

Searches in the MHA directory [42] were performed at the beginning of the MyCyFAPP project (February 2016) and provided more than 500 apps for CF or nutrition management. Of the total, 450 were general purpose apps for managing chronic conditions from which we selected the apps targeted at nutrition and CF-specific apps based on the inclusion and exclusion criteria. Out of them, 9 were specifically targeted at nutrition, 4 were CF-specific games, and 12 were CF-specific apps (Multimedia Appendix 2).

Relevant findings that summarize related aspects in CF-specific mobile apps on the market were classified according to the data extraction criteria (Multimedia Appendix 3). The majority of the apps (19/21, 90%) were provided by individual developers (small to medium enterprises, individuals). Few apps (2/21, 9%) were promoted by the pharma industry (*Cf MedCare* and *MedSched*). One of them, oriented to patients, has very limited features and poor design, and one, oriented to health care professionals, has a focused content in genes and mutations. Only two apps (2/21, 9%) were promoted by hospitals (*Tools4U* and *Genia*), one app (1/21, 5%) by a patient association (*Flower Breath*), and one implemented by the IT department of a hospital from the NHS Tayside region (*Cystic Fibrosis: A Pocket Guide*).

Many of the analyzed apps focused on scientific information and content for education. In these cases, the content was tailored not to patients but to researchers. None of the apps supported pancreatic enzyme replacement therapy (PERT). Most of the analyzed CF-specific apps did not have a concrete business model to ensure sustainability. In Table 2, we summarize the relevant variables by comparing specific and nonspecific CF apps. Download payment ranged from 0 to 2 Euro (US $2.26), and 2/21 apps (9%) included advertisements as a revenue channel.

For non-CF specific apps, business models were much more consolidated, offering coaching services related to calorie intake and healthy nutritional habits. CF-specific apps were focused on the management of the disease (medication and pulmonary symptoms). All of the analyzed apps provided functionalities for recording aspects related to the disease such as medication, meals, and body measurements (eg, weight, forced expiration volume). In addition, some apps provided reminders and educational material. Only two apps provided a feature for communication with doctors and a virtual social community to get in touch with other patients. Nutrition apps were characterized by providing a wide range of features tailored to the user. Features for coaching, personalizing goals, and aspects of food management were predominant in these apps. Only one app was endorsed by international organizations. Most apps are available for both Android and iOS (except for the United States and Sweden, where only iOS versions are available).

Overall, CF apps showed different results in the observed metrics. Reviews and downloads were lower for CF apps than for nutrition apps, as nutrition apps were used by a wider range of users including healthy people and patients with chronic conditions. CF apps were less appealing and usable than nutrition apps (2.66 [SD 1.15] versus 4.01 [SD 0.90], *P*<.001, *z*-value: –2.6). Updates of apps did not include features for the specific management of CF patient needs, such as nutritional counseling, therapy advice, and health literacy.

**Table 2:** Comparative analysis of available apps for cystic fibrosis management and nutrition.

Characteristic		CF^a^-specific apps (n=11), n (%)	Nutrition apps (n=9), n (%)	*P* value	*z*-value
**Licensing**				.19	6.0
	Free	8 (72)	0 (0)		
	Payment	3 (27)	2 (22)		
	Freemium^b^	1 (1)	7 (78)		
**Targeted user**				.20	6.0
	Children	2 (18)	1 (11)		
	Adults	6 (54)	8 (89)		
	All	3 (27)	0 (0)		
Number of reviews, range		5-2160	0-55,000	<.001	–1.6
**Number of downloads**				.26	11.2
	1-100	0	0		
	100-1000	4	1		
	1000-100,000	3	4		
	100,000-1,000,000	1	3		
	1,000,000-5,000,000	0	1		
Last update, aggregate		2017	2018	—^c^	—
Usability score, mean (SD)		2.66 (1.15)	4.01 (0.90)	<.001	–2.6

^a^CF: cystic fibrosis.

^b^Freemium model combines free basic services and advanced payment services.

^c^Not applicable.

## Discussion

### Principal Finding 1: Homogeneity of Health Care Systems in Pediatric Cystic Fibrosis

Routine care was quite homogeneous in Western Europe, based on regular follow-up every 2 or 3 months with full assessment of patients with CF. Treatments were fully covered in almost all countries. Only Belgium and the Netherlands were partially funding the expenses related to treatments. Only Belgium had not established a program for newborn screening for CF. The fact that the visits are scheduled at similar intervals does not mean a similar care delivery, as some are more focused on gastroenterology and nutrition and others more on pulmonology.

### Principal Finding 2: Heterogeneity of Clinical Teams in Pediatric Cystic Fibrosis

Generally, the type of staff involved in CF units includes pediatricians for gastroenterology and pulmonology. Most of the units also included dietitians/nutritionists and nurses. A few units included social workers, psychologists, physical rehabilitators, and pharmacists.

### Principal Finding 3: Apps for Cystic Fibrosis Partially Meet User Needs

Apps for CF were focused on the management of medication, meal intake, pulmonary symptom recording, and education. Our systematic review concludes that current CF apps only partially meet the user needs identified by Floch et al [21], PERT management was not featured in any of the discovered apps, and communication with peers and doctors was implemented only in two apps. From the promoter point of view, the presence of the pharma industry in the app market was limited to one company with a relatively weak presence. Many tools were promoted by individuals and small companies without links to health care systems or CF units. Associations of patients were another group of entities that promoted apps, but once more, their presence was limited and normally their operation range was in the scope of a country or a region. Finally, the most interesting promoting entity was a health care provider, namely a hospital that used the app as a tool for improving care processes for its patients, although support was still limited in terms of available features. CF apps yielded a higher representation in the free licensing business model, whereas nutrition apps showed higher distribution in pay-per-use and in-app purchase business models, although this different behavior was not statistically significant. We evaluated the usability of apps by means of an ad hoc questionnaire and the analysis of three human-computer interaction experts. App usefulness, meaning the ability of the app to cope with actual user needs, is an important attribute but difficult to measure without involving users. We therefore included as surrogate parameters the number of reviews and number of downloads as recorded in MHA at the time of the review.

### mHealth in Pediatric Cystic Fibrosis Management: The Time Is Now

Care models in Western European countries differ slightly in the management of CF, and some of them provide a favorable context for support of self-management. Nutrition is a key factor in the progression of any disease [43], and CF standards of care guidelines recommend nutrition counseling and support at any stage of the disease by expert dietitians [44]. The interviewed units included nutritionist or dietitians on the clinical team, while other disciplines (eg, social workers, psychologists, endocrinologists) weren’t represented in all units. Mobile apps could fill this gap by delivering psychosocial advice and follow-up and, in parallel, offering services for multidisciplinary control by means of distributed technology (external services or services from other countries).

Complete patient empowerment supporting shared decision making (ie, patient is involved in health care decision-making processes) and self-determination (ie, patient has the power to choose own goals) is unrealistic in the case of CF [21]. We do not see mHealth as a unique tool for managing PCF but as a combination of self-management and patient empowerment with telemedicine tools for accessing medical, nutritional, and psychological services irrespective of patient location. This model has been already tested in other chronic diseases in children (eg, type 1 diabetes mellitus), in which a combination of self-reporting outcomes, communication and tools for education, and gamification has shown positive health impact [22]. In a recent systematic review of 23 mHealth interventions, Marcolino et al [11] discovered that the most popular mHealth intervention was based on strategies to promote behavior change. In combination with our results, this shows that the potential of mHealth in PCF needs further efforts, and apps must go beyond offering management tools to support, educate, and motivate patients to adopt and maintain healthier habits.

CF is a disease for which tremendous progress has been made in the field of medical care [45] but for which costs of care are very high and in the case of nonhospital costs can account for up to 50% of total lifetime expenditures of the disease [46]. Children with CF are usually diagnosed in the first year of their life [47] and subsequently need intensive support from family and health care services. In a study on the effect of deprivation, Taylor-Robinson et al [48] discovered that children with CF from more disadvantaged areas had worse growth and lung function compared with children from more affluent areas. Disadvantaged families had a higher burden of treatment, leading to a disruption of school and family life. In this context, telemedicine and mHealth can ameliorate the access to health care services and follow-up by means of remote consultation, access to health education, and patient empowerment [49,50]. Equality in access to health care services has been recognized as a key factor to guarantee equal medical assistance and well-being for all European citizens [51].

The current approach for PCF management is focused on reacting to the effect of health deterioration, focusing on the acute phase of the disease. Democratization of technology is a good opportunity to change the health care management model with the essential objective of improving the quality of life by shifting the hospital-centered approach to a patient-centered paradigm. A better understanding of disease mechanisms and treatment effects leads to an improvement in health outcomes [52]. This suggests that small changes in children’s environment can have large effects on behavior and can be used in self-management of PCF.

The introduction of telemedicine will require changes in health care systems and CF units, but before organizational and cost-effectiveness studies are conducted, further research is needed on the implementation of systems that cover specific patient needs and engage patients and relatives to use the technology for managing the disease.

CF apps including the features of nutrition and PERT self-management have great potential to improve the prognosis of the disease as nutrition is an action that occurs every day, several times a day. If we achieve an adequate nutrient intake and PERT dose, the nutritional status will very likely improve. The importance of this is that nutritional status is the parameter that most correlates with disease prognosis and survival.

### Limitations

Our study has some limitations. Part 1 covered a wide proportion of European countries, although not all of them have been included. Data were collected with the help of the ECFS Patient Registry [29], but data coverage is relatively low in some countries, meaning that not everyone with CF is represented in the data. Moreover, the study has been limited to treatment, follow-up, and general organizational information, excluding other interesting indicators such as costs, resources, and macroeconomic indicators.

Our evaluation of the heterogeneity of clinical teams in PCF has a high risk of selection bias as we only used data from 6 PCF units participating in the MyCyFAPP project. Although the information from these units is reliable, these may not be a representative sample of all units in Europe. In the part 2, the systematic review consisted of queries to the MHA directory search engine, and some apps for CF management may have been missed because the search terms didn’t match the description or the metadata. The unique source for the systematic review of apps was the MHA repository [26] because it contains apps endorsed by patient groups and trustworthy developers. A wider search of apps may be conducted in official app stores such as Google Play (Android) and App Store (iOS) to increase the sample size. Finally, for the evaluation of apps we defined a simple score to discriminate design and appeal, although a standard quality assessment tool should be used to perform an in-depth analysis.

### Comparison With Prior Work

mHealth is a big opportunity to relieve the burden of health care systems [12]. Apps are increasingly being used to follow treatments, improve patient adherence [53], and provide remote access to health services [54]. Prior research has focused on the potential of mHealth in the self-management of diseases [23], but the potential of mHealth to meet the existing needs of patients using existing app functionalities should also be considered.

A mobile app represents a unique channel for the direct communication between patients and doctors beyond classical mechanisms like phone calls or emails. Health care professionals will have to adapt their clinical protocols and internal procedures to this new paradigm [24]. More evidence is needed on the impact and effectiveness of integrating such tools into CF routine care [55]. Wearable sensors and tracking systems [56] are opportunities for the automatic collection of data by CF-related apps, a factor that could increase their effectiveness in follow-up, gamification, and motivation.

An explosion of health-related apps has appeared in the different app markets in the last 5 to 7 years [11]. New apps are landing not in an incipient research and market scenario but in a consolidated research field and business. Apps in health should demonstrate their effectiveness in meeting clinical and engagement end points in the long term [57]. Multidisciplinary research should aim at delivering tools for remote and self-management from several perspectives, including usability aspects, motivation factors, perceived usefulness, and economic sustainability [58].

### Conclusions

The health care context for PCF is a good opportunity for the development of mHealth solutions. The heterogeneity of clinical teams, coverage of national health systems, and geographical distribution of CF units build a suitable scenario for the use of mHealth solutions. CF apps included in our study are mainly based on symptom recording and management of reminders for medications and meals. mHealth features for specific nutritional management, social support, communication with health professionals, and decision support should be explored to increase the adoption of apps in the management of CF.

## Appendix

Multimedia Appendix 1 Description of the characteristics of each referenced pediatric cystic fibrosis center.

Multimedia Appendix 2 Diagram of searches and selected apps.

Multimedia Appendix 3 List of analyzed apps and their features.

## Abbreviations

CF: cystic fibrosis
ECFS: European Cystic Fibrosis Society
MHA: myhealthapps.net directory
mHealth: mobile health
NSCF: Norwegian Resource Center for Cystic Fibrosis
NHS: National Health System
PCF: pediatric cystic fibrosis
PERT: pancreatic enzyme replacement therapy
