# Supplementary material for: The Potential of Self-Management mHealth for Pediatric Cystic Fibrosis: Mixed-Methods Study for Health Care and App Assessment
Source: JMIR Mhealth Uhealth. 2019 Apr 18;7(4):e13362. doi: 10.2196/13362 (PMC6495294; doi:10.2196/13362)
Supplement: Multimedia Appendix 1 [file mhealth_v7i4e13362_app1.pdf]

Multimedia Appendix 1. Description of the characteristics of each referenced pediatric CF center.

**Country (Hospital unit)**	**Paediatric Cystic Fibrosis unit**	**PCF Population Covered (*)**	**Clinical Staff (**)**									**Treatment and follow-up**	**Access and Coverage**
			PG	PP	EN	ND	NR	PS	PR	SW	PH		
Spain (La Fe-Valencia)	Yes (different from adults`)	105	2	2		1	2		1			At least every 3 months in Gastroenterology and Pulmonology Services	Full coverage. Access in specialized care.
Spain (Ramon y Cajal Hospital - Madrid)	Yes (integrated in the Paediatric Dept.)	148	2	3	2	2	2	1	1			Every 2-3 months in the CF unit (at least, 6 visits per year) in Gastroenterology and Pulmonology Services.	Full coverage. Access in specialized care.
Italy (USMilano)	Yes	399	4	3		2	1	2	4	1		Every 2-3 months in the CF unit (but depends on patient status, can be daily or each 6 months) in Gastroenterology and Pulmonology Services.	Full coverage. Access in specialized care.
Portugal (AIDFM)	Yes	60		2		3	2	1				Every 1-2 months, in the CF unit (depending of patient status) in gastroenterology and pulmonology Services. Follow up includes dietician (with a nutritional evaluation) and nurse (adherence to medical therapy).	Full coverage. Access in specialized care.

Belgium (KU Leuven Hospital)	No (integrated with adults)	103		4		3	4	1	4	2		Every 2-3 months by the whole team	Reimbursement for part of the multidisciplinary team care. Reimbursement of part of the medication (including enzymes and vitamins)
Netherlands (Erasmus MC Rotterdam)	No (integrated in the university hospital)	150	6	2		2	2	2	2	1	1	Follow up at least every 3 months by the pulmonologist, and at least once a year by the paediatric gastroenterologist.	Medication payment through insurance.

(*) As June 2017. (**) Clinical Staff can include: Paediatric Gastroenterologist (PG), Paediatric Pneumologist (PP), Endocrinologist (EN), Nutritionist-dietitian (ND), Nurse (NR), Psychologist (PS), Physical Rehabilitator (PR), Social Worker (SW) and Pharmacist (PH).
