# Supplementary figures and images for: The Potential of Self-Management mHealth for Pediatric Cystic Fibrosis: Mixed-Methods Study for Health Care and App Assessment
Source: JMIR Mhealth Uhealth. 2019 Apr 18;7(4):e13362. doi: 10.2196/13362 (PMC6495294; doi:10.2196/13362)

## Appendix 2

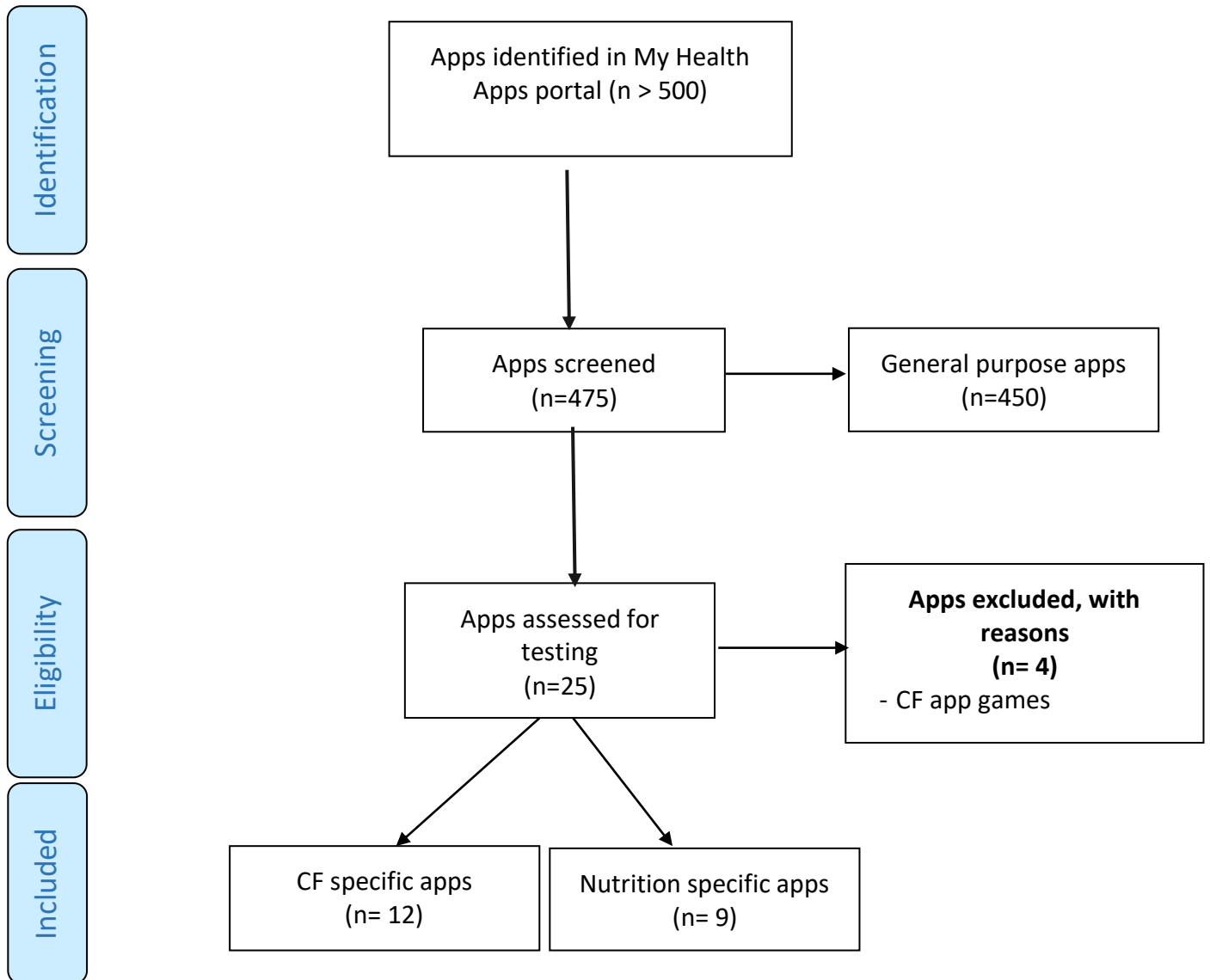

Supplement: Multimedia Appendix 2 [file mhealth_v7i4e13362_app2.pdf]
