# Supplementary material for: The Potential of Self-Management mHealth for Pediatric Cystic Fibrosis: Mixed-Methods Study for Health Care and App Assessment
Source: JMIR Mhealth Uhealth. 2019 Apr 18;7(4):e13362. doi: 10.2196/13362 (PMC6495294; doi:10.2196/13362)
Supplement: Multimedia Appendix 3 [file mhealth_v7i4e13362_app3.pdf]

Multimedia Appendix 3. List of analysed apps. Name, promoter and characteristics of the app. CF/Nutrition: specific app for general CF management or Nutrition management. PERT: Pancreatic Enzyme Replacement therapy dose calculator. Med: Drug therapy organisation. Diet: Nutrition management. Diary: Record of symptoms. Edu: Educational material. Goals: Enables setting up goals. Advice: Provides tailored advice or coaching. COM: Communication with doctors and communication with peers. AirCle: Provides physiotherapy or airway clearance. (*) Only for visual feedback of Flutter® device.

**Name**	**Promoter**	**CF/ Nutrition**	**PERT**	**Med**	**Diet**	**Diary**	**Edu**	**Goals**	**Advice**	**COM**	**AirCle**
Cf MedCare	Vertex Pharma	CF		X		X		X		X	
Tools4U	Nationwide Children's Hospital	CF			X	X	X				
MyCF	Individuals	CF		X		X					
CF Notebook	Ocean Vector	CF		X	X	X					
My Fight Against Cystic Fibrosis	Larry B. King	CF					X				
MedSched	Blue Diamond	CF		X							
MyTherapy	Smartpatient GmbH	CF		X							
Flower Breath (*)	Defitech foundation	CF									X
Genia	Genia	CF					X			X	
Bubble Wizard	Pak (Company)	CF					X				
Cystic Fibrosis: a pocket guide	NHS Tayside, Univ. Dundee	CF					X				
CF GeneE	Vertex Pharma	CF					X				
Kurbo	Kurbo Health Inc	Nutrition			X			X	X		
Yazio	Yazio, DE	Nutrition			X			X	X		
Lifesum	Lifesum, US	Nutrition			X						
Nutrino	ComoComo, ES	Nutrition			X			X	X		
Ultimate food value dairy	Fenlander Software Solutions, US	Nutrition			X						
Nutrition Menu	Shroomies	Nutrition			X						

Nutrition tracker PRO	BioStatusHealth	Nutrition			X				X		
Food tracker PRO	Aspyre	Nutrition			X			X			
Rise	Rise Labs	Nutrition			X			X	X		
